# Perceptual distortions characteristic of Alice in Wonderland syndrome in contemporary figurative painting

**DOI:** 10.3389/fpsyt.2024.1466666

**Published:** 2024-12-04

**Authors:** Erica Hyatt, Jan Dirk Blom

**Affiliations:** ^1^ Faculty of Social and Behavioural Sciences, Leiden University, Leiden, Netherlands; ^2^ Outpatient Clinic for Uncommon Psychiatric Syndromes, Parnassia Psychiatric Institute, The Hague, Netherlands; ^3^ Department of Psychiatry, University of Groningen, Groningen, Netherlands

**Keywords:** angor animi, art, hallucination, metamorphopsia, mimesis, realism, synesthesia, visual perception

## Abstract

**Background:**

Alice in Wonderland syndrome (AIWS) is a neurological condition characterized by perceptual distortions, most of which are visual in nature (metamorphopsias). Over the past decade there has been a movement in contemporary figurative painting away from strict mimesis toward depicting distortions of the painting’s subject, called disrupted realism. In certain cases the similarities between the distortions in those paintings and those characteristic of AIWS are so striking that we suspect that artists may have experienced distorted perceptions themselves and used them for creative inspiration.

**Methods:**

To empirically test this hypothesis we interviewed 20 painters who frequently use distortions in their work, using the SIntAD, a tailor-made, semi-structured questionnaire. We then carried out a phenomenological analysis of the perceptual phenomena reported on, and compared them with those in their paintings.

**Results:**

Of the artists interviewed, 85% reported on having experienced positive disorders of perception in general (comprising hallucinations, perceptual distortions and other perceptual phenomena), with 55% reporting on a total number of 15 metamorphopsias. Most artists had not been aware of having these distortions to their perception. Nonetheless, most did not use these specific distortions in their work, but rather different types.

**Conclusion:**

Symptoms of AIWS and other positive disorders of perception are common among contemporary painters who frequently use distortions in their figurative work, although perhaps not more common than in the general population. Artists in the disrupted-realism movement tend not to mimic their own perceptual distortions in their work, although they do feel inspired to distort their work in different ways.

## 1 Introduction

Plato famously stated that art imitates life. In his theory of mimesis he suggested the closer a work of art approached truth, the more beautiful it would be. Since the ancient Greek philosopher also held that our perceptions are mere shadows of existence, and art is therefore always ‘twice removed from the truth’ (1), this observation takes on a new meaning in the context of distorted realism, a movement in contemporary art where realistic figurative paintings show distorted aspects reminiscent of Alice in Wonderland syndrome (AIWS). We here ask ourselves whether artists in this new artistic movement paint what they see, or merely add distortions to their work for aesthetic or even commercial reasons. We moreover ask ourselves whether those who paint what they see are aware that their perceptual distortions are characteristic of AIWS.

### 1.1 Alice in Wonderland syndrome

**Figure 1:**
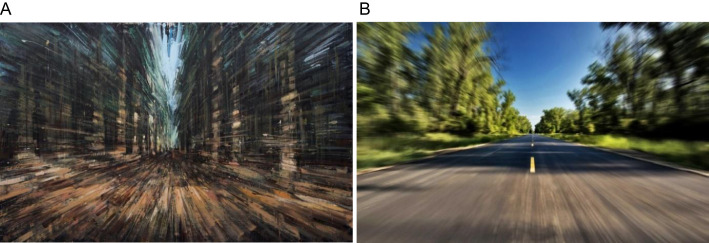
**(A)** *Biking in White’s Woods*, oil on panel by V. D’Ospino; **(B)** image used in clinical practice to explore the presence of porropsia (i.e. seeing stationary objects continually moving away).

AIWS is a neurological condition characterized by phenomena known as perceptual distortions (2, 3). These differ from hallucinations (where something is perceived that is not there) and illusions (where an existing object is perceived as something else) in that existing objects are perceived such that one or more specific aspects appear to be different, without any consequences for their gestalt. Some common examples are micropsia (seeing things smaller than they are), macropsia (seeing things larger) and prosopometamorphopsia (seeing changes to people’s faces). These are all examples of *visual* distortions (i.e. metamorphopsias), but the distortions in the context of AIWS can also be experienced in the somaesthetic, auditory, and temporal modalities, and occasionally in the olfactory and gustatory modalities (4). The visual ones are most common though, and it is to this group that we refer in relation to the striking similarities that we found in certain contemporary figurative paintings. For example, Valerio D’Ospino’s painting *Biking in White’s Woods* (**Figure 1A**) bears an uncanny resemblance to an image (**Figure 1B**) used diagnostically by some specialists when diagnosing distortions to the perception of movement, more specifically porropsia, a perceptual distortion where stationary objects appear to be moving away (5).

### 1.2 Disrupted realism

D’Ospino’s painting was included in an exhibition titled *Disrupted Realism* held in Philadelphia’s Stanek Gallery in 2018 with a book of the same title published shortly thereafter (6). Disrupted realism, figurative abstraction, distorted artwork; a variety of names are used to describe this contemporary art movement, although distortion in art is not new of course. We find it in cubism, surrealism, expressionism, impressionism, and numerous other schools. One calls to mind the unusual faces in Picasso portraits, Dalí’s melting objects, Giacometti’s elongated figures, Monet’s colorful haystacks. What is novel are the distortions arising in realist work, which had historically aimed to create an accurate depiction of the external world, in conformity with Plato’s *mimesis*. Artwork that is expressive or abstract can be interpreted and critiqued. We can discuss the artist’s intention or the deeper meaning of the work. But when we turn to realism, the possibility opens to explore what the artist actually perceives.

### 1.3 Historical examples

**Figure 2:**
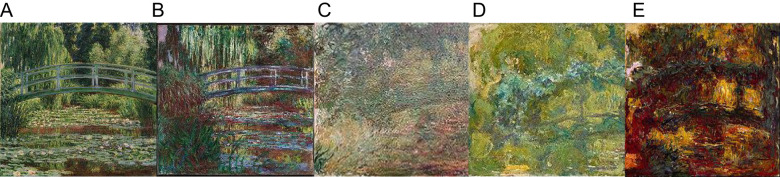
Paintings of the same scene by Claude Monet over the years: **(A)** *The Japanese Footbridge and the Water Lily Pool*, 1899; **(B)** *Water Lily Pond*, 1900; **(C)** *Japanese Bridge* (detail), 1918; **(D)** *La Passerelle sur le Bassin aux Nymphéas*, 1919; **(E)** *The Japanese Footbridge*, 1922.

The well-known impressionist, Claude Monet, was diagnosed with cataracts in 1912. The nuclear cataracts which he suffered from cause altered color perception (7). Since he painted the same subjects over the course of decades, we can compare his paintings from earlier years with those painted as his vision worsened to near blindness from his diagnosis in 1912 until his first eye surgery in January of 1923. Thus Monet’s oft-depicted Japanese footbridge allows us to see the progression of his eye disease, with early representations capturing nuanced greens and subtle shades of pink and blue, and later works skewing toward brown, yellow and muddy reds as his vision worsened (**Figure 2**).

Edgar Degas’ deteriorating vision is also well documented, his later works revealing his failing eyesight as edges become blurred and less defined, and figures lack detail (7, 8). This research sets a precedent for using an artist’s work to gain insight into their perceptions (7–10). AIWS is mainly concerned with neurological causes of perceptual distortion in a bottom-up fashion, although sometimes peripheral conditions may also play a role. Its characteristic distortions arise mostly from the brain’s failure to detect and represent highly specific incoming sensory signals. Such problems in visual perception caused by eye disease and central problems in perception distort the way people experience their reality. Just as Monet’s cataracts caused his perceptions to change in concrete and predictable ways, his world darkening, blurring and shifting toward brown and muddy colors, metamorphopsias with a central cause can also be categorized (3). A well-known category is dysmorphopsia, a distortion of the contours of objects that makes them appear wavy (3). Safran et al. (11) discuss the presumed dysmorphopsia experienced by the artist, Francis Bacon. A thorough review of Bacon’s oeuvre reveals that he had no difficulty in rendering objects comprised of straight lines, such as doors and beds. His distortions seemed to quite specifically arise when he painted people’s faces. The suggestion that he suffered from prosopometamorphopsia therefore seems more fitting (12). Bacon’s disturbing yet fascinating paintings have drawn much attention and discussion from art critics, connoisseurs and philosophers, and more recently from those interested in neuroesthetics and neurology (11, 13). There is no question that they make a strong impression on the viewer. Former UK prime minister Margaret Thatcher dismissed him as ‘the man who paints those dreadful pictures’ (14) yet despite the strong negative reactions his paintings inspire, they remain curiously alluring. The attraction of artwork that depicts an artist’s unique impression of their world along with the distortions they perceive may even be appealing *because* of those distortions.

### 1.4 AIWS in the general population

There are no systematic studies on the incidence and prevalence of AIWS in the general population. However, even though the syndrome is diagnosed quite rarely in clinical practice, there is evidence that experiencing some sort of transient metamorphopsia may be relatively common. A search in the popular press for pieces on AIWS reveals multiple articles highlighting the story of someone suffering from more extreme forms of this condition (15, 16). A newspaper article in the UK’s Daily Mail describes the case of a 24-year-old woman diagnosed with AIWS after watching a popular medical television drama. Her case is typical in that her episodes of perceptual distortion resolved quickly. Most remarkable was the number of people who responded in the comments sharing their own AIWS-like distortions and reporting that family members also experienced metamorphopsias (15). Out of the 119 comments on the article, 52 people had experienced AIWS, and five commenters mentioned that multiple family members also experienced symptoms (**Supplementary Material**, **Supplementary Table S1**). A study examining the prevalence of micropsia and macropsia in several thousand Japanese high school students supports this observation (17). The authors found that around 9% of the students interviewed had experienced a transient and self-limiting symptom typical of AIWS within the past six months (17). A Finnish study among 297 slightly older people even found rates of up to 38% for symptoms of AIWS (18). Considering that transient metamorphopsias associated with AIWS are likely more common than originally suspected, many viewers of paintings with distortions may recognize something hauntingly familiar in the images depicted. This may explain their fascination with this type of art, without realizing why, and without knowing the reasons why the artist decided to add these distorted aspects.

In this article we explore what types of distortion can be discerned in contemporary figurative paintings in the so-called disruptive-realism movement and whether they comply with the metamorphopsias considered characteristic of AIWS. In addition, we explore the reasons that artists give for applying such distortions to their work and whether they ever experienced symptoms of AIWS themselves.

## 2 Methods

We collected a convenience sample of 20 contemporary figurative painters who consistently create distorted representations in their work. Artists were recruited from online social media platforms, via image searches for distorted artwork, and from John Seed’s book *Disrupted Realism* (6). Recruitment took place from March, 2022, through September, 2023. We restricted our investigation to living artists so that they could be interviewed. The distortions in the artwork were categorized and compared with defined perceptual distortions described in the literature on AIWS using a classification of metamorphopsias (3). The artists were interviewed with the aid of a tailor-made, semi-structured questionnaire. They all provided recorded verbal consent. Ethical approval was not required since the research relies exclusively on the information provided by healthy individuals and the secondary use of anonymous and aggregated information. An exception to this are the painters whose works are here reproduced, who kindly provided informed consent for this and are aware that these works may give clues to their identity. Given the relatively small numbers, we considered the use of statistics of no additional value.

### 2.1 Interviewing the artists

Artists were interviewed by the first author using a purpose-built, semi-structured, non-validated questionnaire called the Semi-Structured Interview for Artists using Distortions in Painting (SIntAD; **Supplementary Material, S2**). The questionnaire consists of 11 questions divided into three sections. Section 1 is composed of open-ended questions about the artist’s style, artistic intention, and their perceptions. Section 2 is about the reasons that artists give for using distortions in their work. Section 3 contains follow-up questions to capture any perceptual distortions not yet discussed and about continuing communication and contact with the artist. The interviews were held either via telephone or video call and were recorded with the artists’ permission. In three cases, questionnaires were filled out by artists and returned via email to accommodate artists who would not otherwise have participated. Recorded interviews were then transcribed. Data were aggregated in a spreadsheet and analyzed.

## 3 Results

### 3.1 Demographics

The participants (N=20, mean age 46 years, age range 24 to 68 years) were all contemporary representational painters. Gender was quite evenly distributed between male and female (11 M, 9 F) with one biological male identifying as a female.

### 3.2 Distortions in paintings

**Figure 3:**
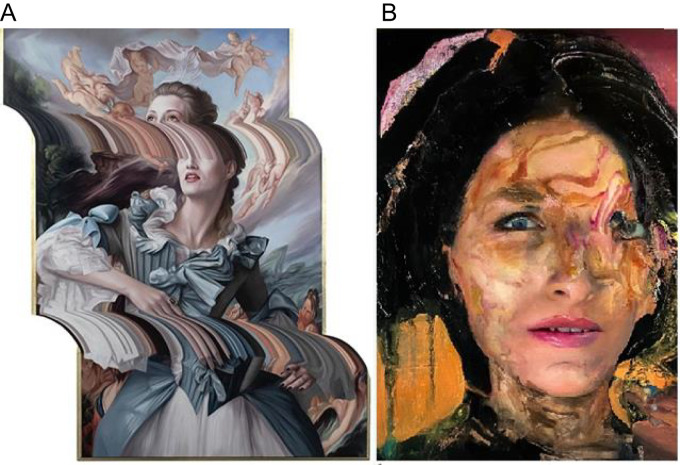
**(A)** *Dying on the Vine*, oil on panel by B. Ashton (2023). **(B)** *The Wisdom*, oil on canvas by C. Westerhout (2022).

Artists who consistently created work which incorporated distortions were asked to participate in the research. Some of these distortions were more technical in nature (**Figure 3A**), recalling glitches one might find in interlaced video, alignment issues in a panoramic photo, or an image altered with image-editing software. Other distortions could be characterized as more organic in nature and are reminiscent of the sorts of perceptions described by people who experience symptoms of AIWS (in this case prosopometamorphopsia; **Figure 3B**).

### 3.3 Symptoms

**Figure 4:**
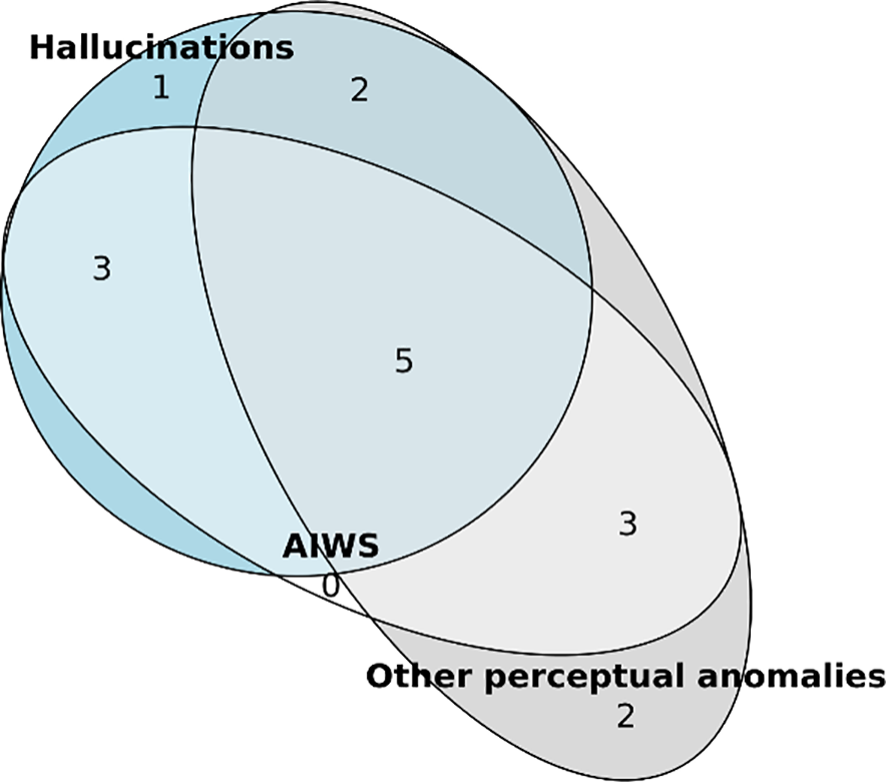
Distribution of perceptual distortions, hallucinations and other perceptual phenomena in a subgroup of the artists interviewed (n=17).

During our interviews we recorded all perceptual anomalies reported by the artists. We then categorized these in accordance with existing medical classifications into perceptual distortions, hallucinations and other positive disorders of perception. Three participants reported no perceptual anomalies. The other 17 reported a variety of phenomena, with five of them reporting all three types (i.e. perceptual distortions, hallucinations and other perceptual phenomena; **Figure 4**). For a complete list of all the 74 symptoms reported on, see the **Supplementary Material**, **Supplementary Table S1**.

#### 3.3.1 Metamorphopsias

**Figure 5:**
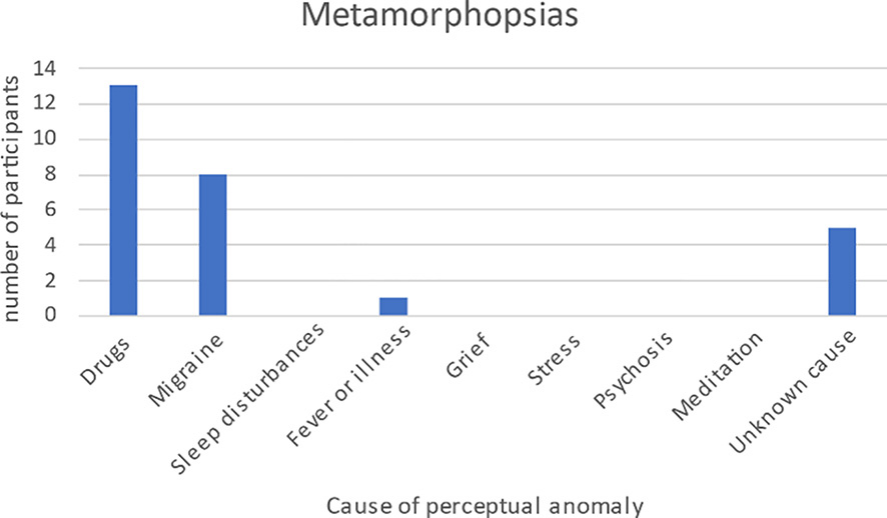
Metamorphopsias and their associations with various conditions.

Among the 74 perceptual phenomena recorded were 15 different types of metamorphopsia, reported on by 11 participants. The most frequent type was hyperchromatopsia, a distortion in which colors are seen as exceptionally vivid and brilliant. This was reported four times. Enhanced stereoscopic vision was reported three times. All other metamorphopsias that were reported were reported only once (seven types) or twice (five types). We did not systematically record the duration of symptoms, but all but two were fleeting or temporary in duration. The only enduring symptoms reported were depersonalization (see Section 3.3.3) and a case of whole-body hyposchematia, a perceptual distortion in which a person underestimates the size that their body takes up in external space (**Figure 5**).

##### 3.3.1.1 Sensory modalities involved

**Figure 6:**
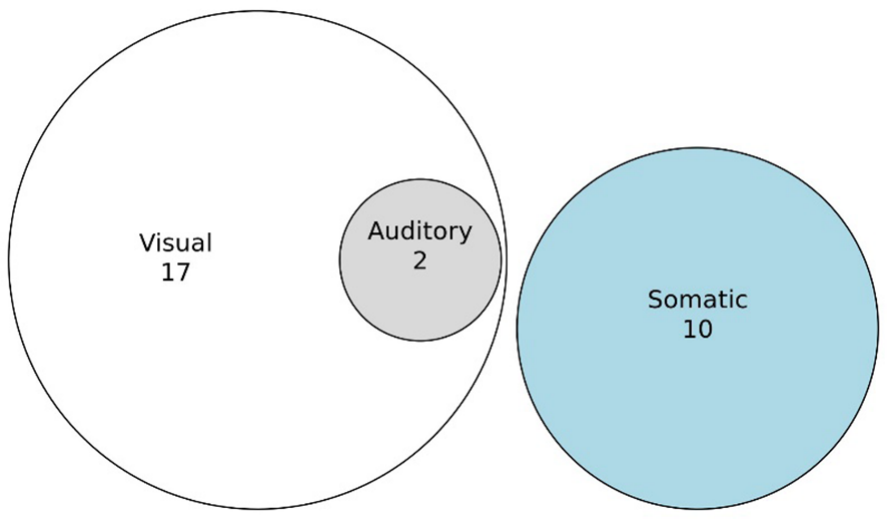
Sensory modalities in which perceptual distortions were experienced.

Currently over 60 symptoms fall under the umbrella of AIWS. In our sample, 13 of our 20 participants reported between one and four AIWS symptoms. When possible we classified reported distorted perceptions as auditory, visual, somaesthetic, temporal, or the appropriate combination of these modalities. There were 19 reports of visual distortions, two of which had an additional auditory component, and 10 reports of somaesthetic distortions (**Figure 6**). Macro- and microsomatognosia and hyposchematia were included in the somaesthetic modality. There was one participant who had experienced time distortions. We had no reports of gustatory or olfactory distortions. There was also one person who experienced complicated metamorphopsia of a sense of impending doom (angor animi) 12 hours prior to a heart attack (see also Section 4.7). Although angor animi is not included in current classifications of AIWS, it is noteworthy that Todd did mention it in his original paper on AIWS (2).

#### 3.3.2 Hallucinations

**Figure 7:**
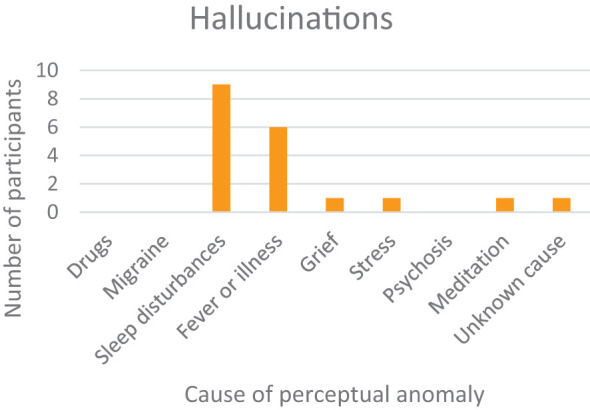
Hallucinations and their association with various conditions.

Our open questions regarding positive disorders of perception yielded interesting additional information. Eight participants reported hallucinations, although most of these were sleep-related. The reported phenomena were comprised of hypnagogic and hypnopompic hallucinations, auditory hallucinations at sleep onset, sleep-deprivation hallucinations (visual), a dead-weight hallucination upon waking (a somatosensory hallucination where one experiences being pulled down to the ground), and an incubus phenomenon in conjunction with sleep paralysis (**Figure 7**). Six instances of hallucination were experienced during illness or high fever. There was one report of a hallucination in conjunction with grief, one during severe stress, and one experienced at a meditation retreat. Finally, there was one report of an unclassifiable phenomenon, designated by the participant as an unidentifiable flying object (UFO). Since it involved a shared experience, a biomedical explanation might be a collective hallucination.

##### 3.3.2.1 Sensory modalities involved

**Figure 8:**
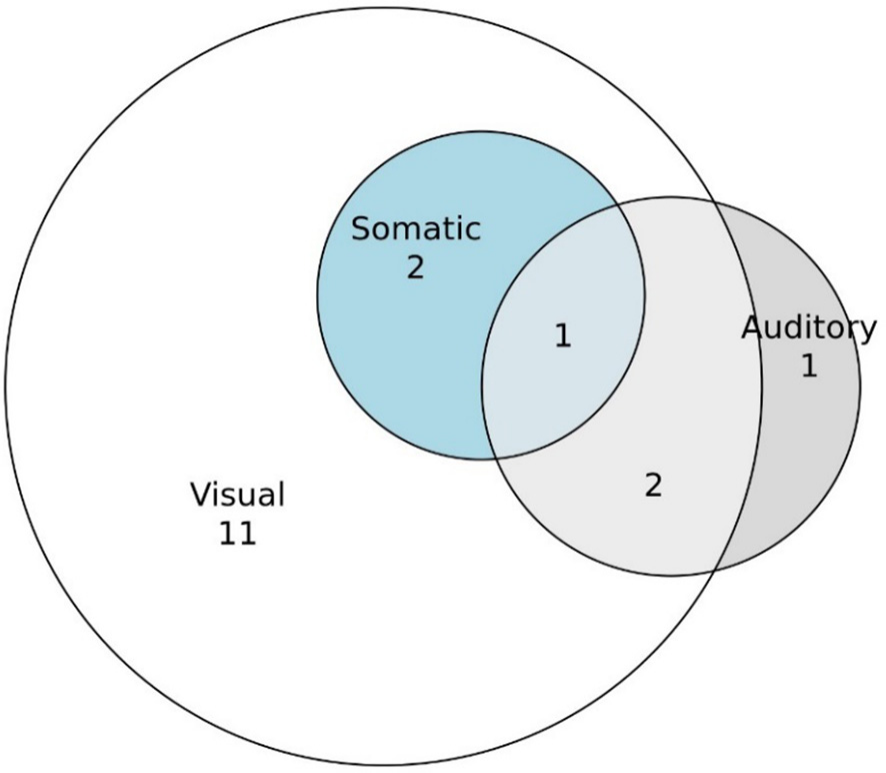
Sensory modalities in which hallucinations were experienced.

As we saw with metamorphopsias, our participants’ hallucinations were predominately visual in nature; 16 of the reported phenomena had a visual component (**Figure 8**). Only three hallucinations had a somatic component, two of which were solely somaesthetic. Only one hallucination was purely auditory in nature, versus three with shared visual and auditory components. Only one reported hallucination was experienced in all of these three sensory modalities.

#### 3.3.3 Other perceptual phenomena

**Figure 9:**
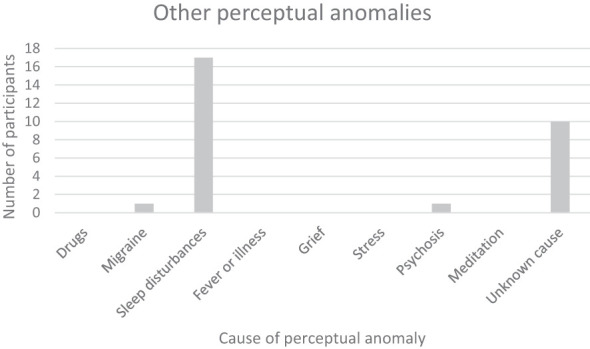
Other perceptual anomalies.

**Figure 10:**
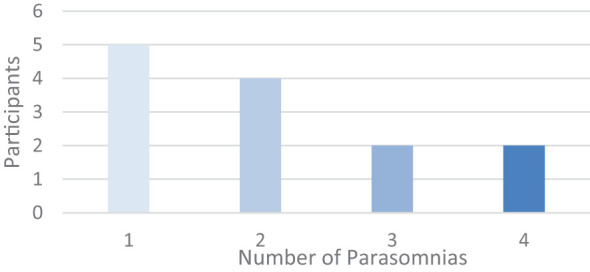
Number of parasomnia reported (n=13).

Four participants reported synaesthesia. Depersonalization and derealization, if counted as one category, were reported three times; in one instance this was lifelong. The other positive disorders of perception that we recorded were overwhelmingly related to sleep disturbances (**Figure 9**). Sleep issues, reported by 13 of our 20 participants, ranged from common phenomena such as insomnia, hypnic jerks, unusually vivid dreams, nightmares and night terrors to symptoms of REM-sleep behavior disorder, out-of-body experiences and the aforementioned hypnagogia and instances of sleep paralysis. The most reported were insomnia (four people), hypnopompic hallucinations (the persistence of dream imagery into waking, four people), and unusually vivid dreams (three people). One quarter of our participants experienced one sleep disturbance of some kind and almost half of the total group reported having more than one sleep disturbance (**Figure 10**).

### 3.4 Distortions in paintings versus distorted perception

All participants were aware that they used distortions in their paintings. As reasons for this five of them stated that they were inspired by their own perceptual distortions, with all five of them mimicking what they actually experienced in at least one piece and sometimes an entire body of work. However, the majority of those who had experienced perceptual distortions (17) used different types of distortion in their work. When asked about this, the five artists who had used a distorted perception as the basis for a painting were aware that the distortions in their paintings might be inspired by the fact that they sometimes perceived things differently. The remaining twelve artists had not considered that experiencing distortions to their perception might increase the likelihood that they bend reality in other ways in their work, but eight found the question intriguing and worthy of more consideration, while two considered it could be an influence, and two didn’t see any connection. One person stated that he had been inspired by other people’s work to use this style, two that they had been inspired by neuroscience and none explicitly stated that they hoped to benefit from this style commercially. Finally, five artists made a direct connection between a distorted perception they had experienced in the context of conditions such as migraine, psychosis, sleep-related conditions, fever and substance use, and the artwork they had created.

## 4 Discussion

Among the 20 painters we interviewed, 17 (85%) reported on 74 different positive disorders of perception. Of them, 11 (55%) had experienced perceptual distortions reminiscent of AIWS. Notable was the number of distortions experienced by some participants. In 85% of the cases people with AIWS experience only one symptom, though it may be experienced repeatedly (4). Here we found that two people (18%) reported on a single distortion, six (55%) on two distortions, one (9%) on three distortions and two (18%) on four distortions. One of the two latter participants experienced hyperchromatopsia and hypoacusis (hearing sounds quieter than normal), and additionally reported that as a child she had experienced macrosomatognosia (experiencing one’s body or a body part as very large), microsomatognosia (experiencing one’s body or a body part as very small) and synesthesia (not a symptom of AIWS), with a three-dimensional, colored perception of music spiraling and fading away. The majority of participants reported having fewer perceptual distortions as they aged. On the basis of their self-reports it was not possible to quantify the rate of decrease, but except for one, all of them reported either isolated incidents in the past or said that the frequency had declined as they had gotten older. In short, on average, the number of distortions and other perceptual phenomena in the group under study was higher than in an average group of people with AIWS.

### 4.1 Direct effects of perceptual distortions on paintings

**Figure 11:**
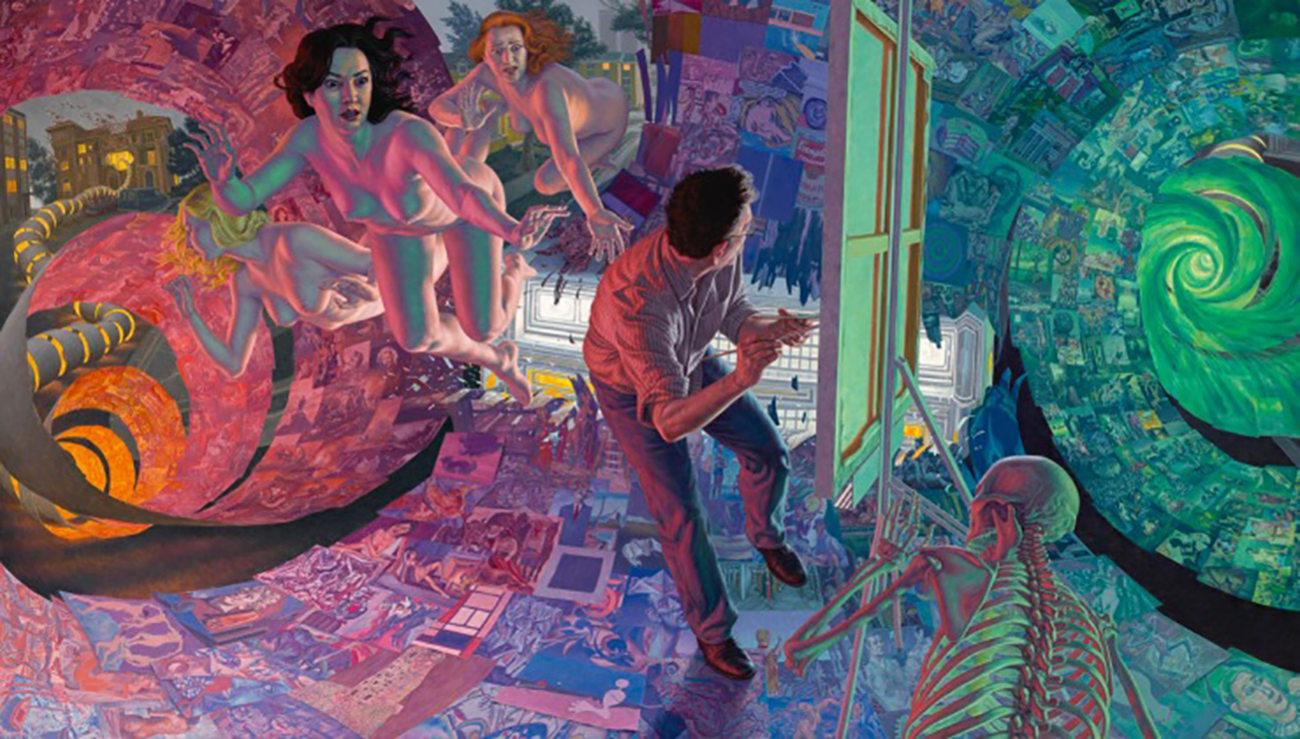
*The Dream of Art History*, oil on canvas by F.S. Hess (2018).

**Figure 12:**
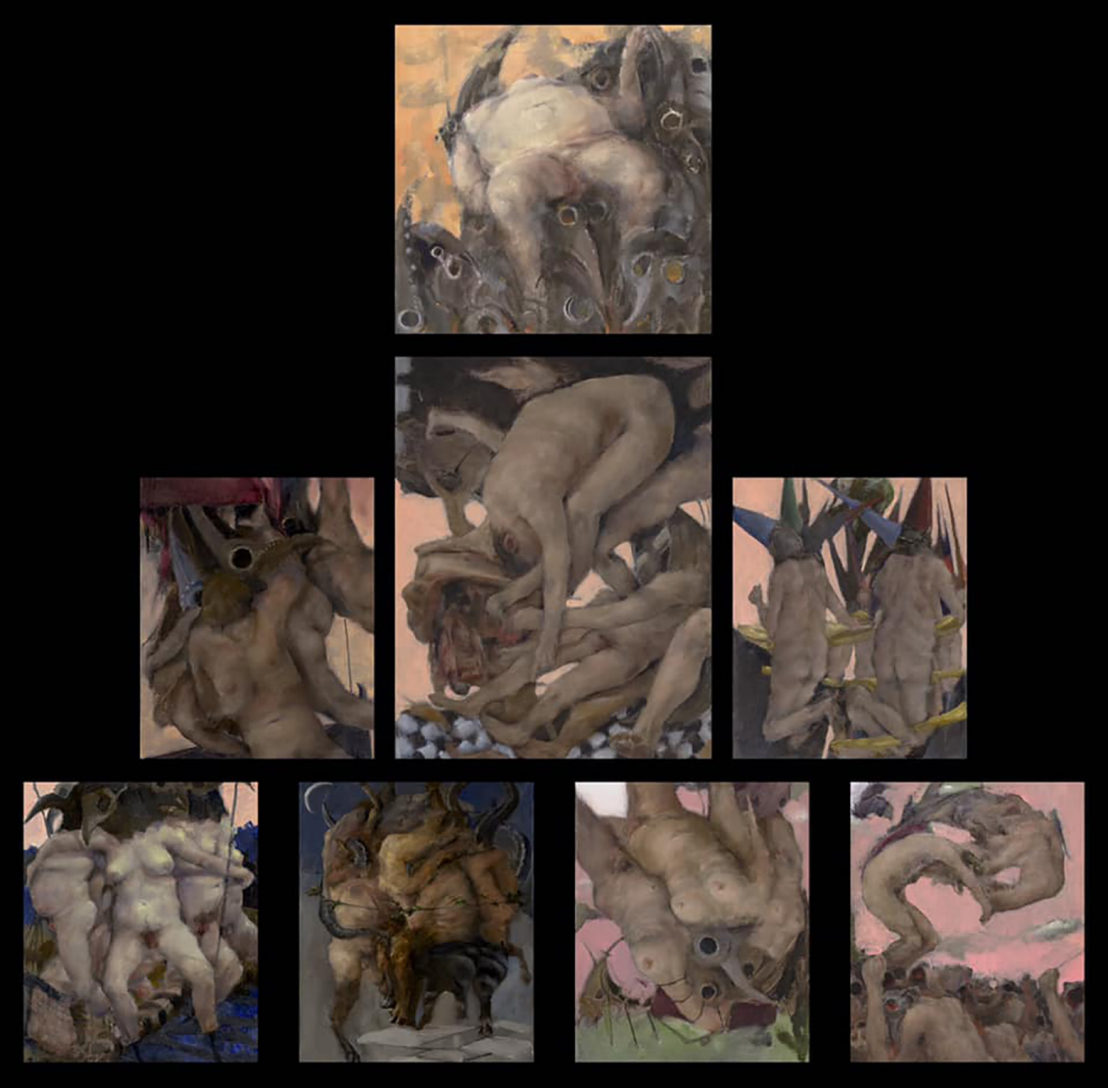
*The Wages of Pestilence and Folly*, oil on canvas panels by K. Kaapcke (2020).

**Figure 13:**
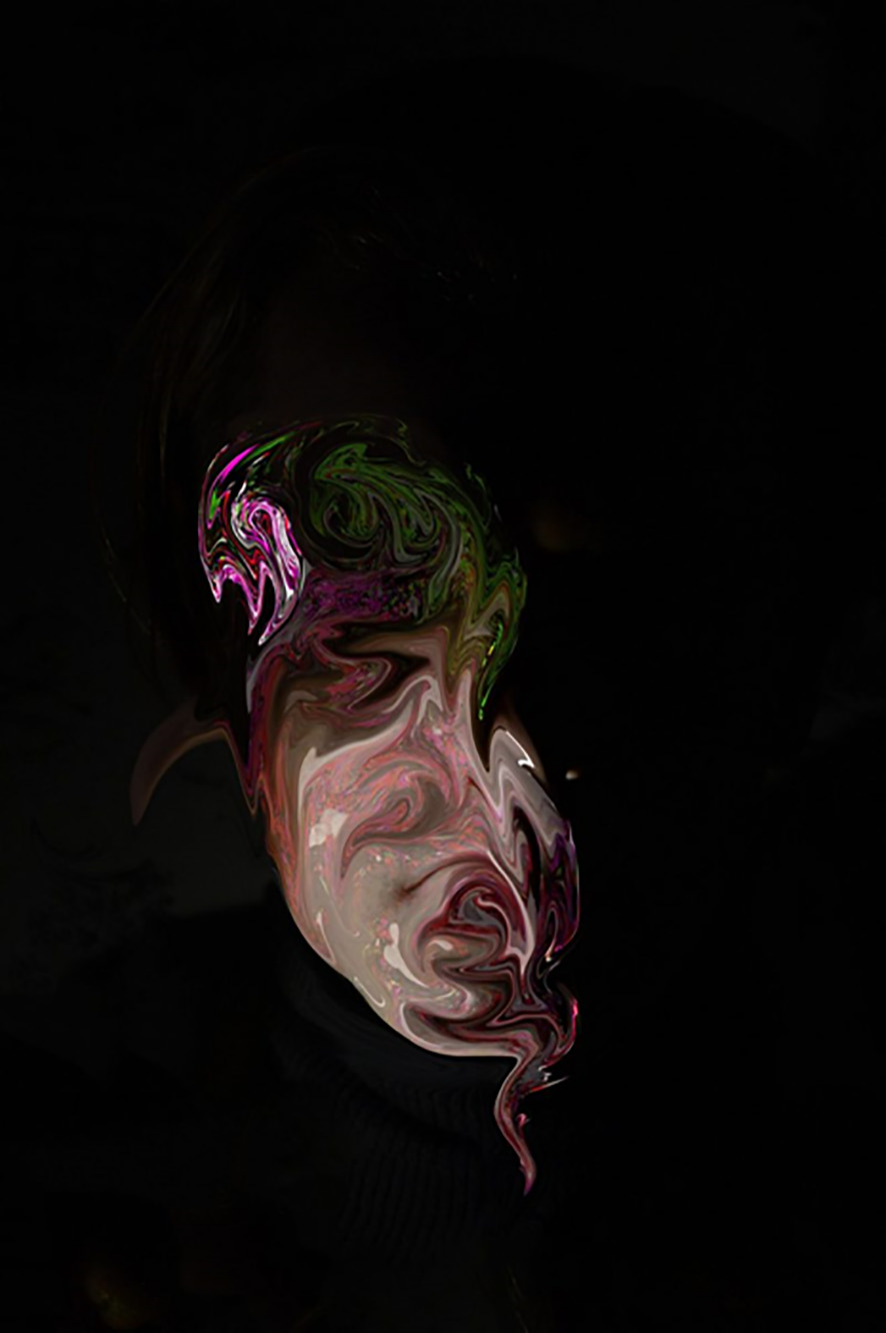
*Light Starts to Melt*, digital artwork by M. Archibald (2020).

As we saw, of the 17 artists who reported experiencing any type of distorted perception, five (29%) had used this explicitly as a basis for a painting. Two people portrayed their distorted perceptions consistently throughout their body of work. One of these artists’ work depicts various aspects of sleep paralysis and related parasomnias, the other consistently paints nebulous, ghostly images of herself in reaction to thoughts of becoming invisible (depersonalization). Two artists painted works inspired by hallucinations experienced during a high fever. One painted a very large figurative work based on a fever hallucination (**Figure 11**). In contrast, his other distorted work uses technology to create a distorted reference image or warping of linear perspective while painting, to thus intentionally challenge the viewer’s perceptions. The second artist painted an entire series of work while experiencing fever-induced hallucinations due to Covid-19, actually painting as she saw the images (**Figure 12**). The other works of these two artists contain distortions, so these paintings depicting hallucinations caused by fever were an exception in their oeuvre. The fifth artist who portrayed her distorted perceptions used a metamorphopsia experienced during a migraine prodrome as inspiration for her work, poignantly titled *Light Starts to Melt* (**Figure 13**). Studies indicate that migraine patients who experience migraine with aura are more likely to experience AIWS symptoms than those without aura (19, 20). Our results support these findings. Of our 20 participants, nine self-reported suffering from migraine, six of which with aura. What is more, all six people who experienced migraine with aura also reported AIWS symptoms.

### 4.2 Indirect effects

**Figure 14:**
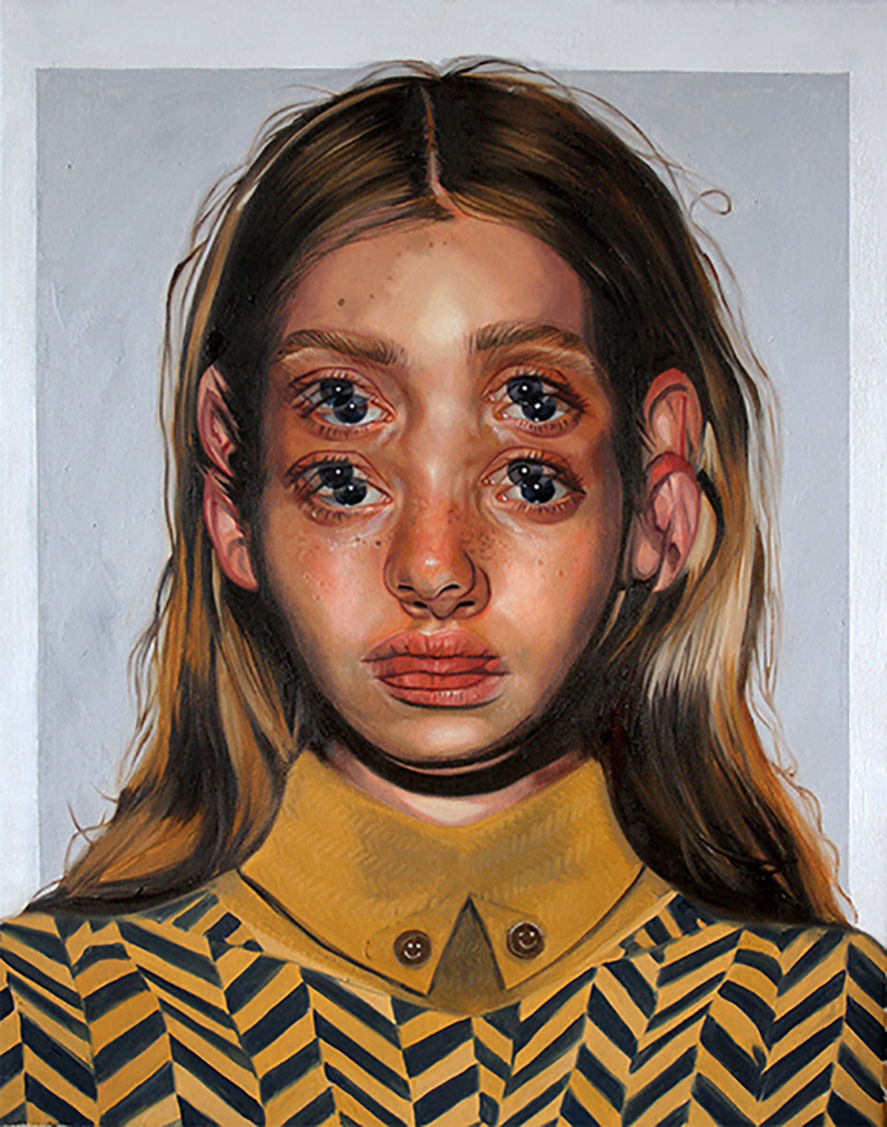
*Entitled*, oil on canvas by A. Garant (2019).

All artists whom we chose to interview were selected because of the consistent portrayal of distortions in their work reminiscent of AIWS. Although 55% had experienced distortions to their perceptions, the distortions seen in paintings were most often not those that they had experienced themselves. One of our participants, selected for participation because of the intensely colorful choices in his work, reminiscent of hyperchromatopsia, did not report any unusual color perception but did report regular episodes of total-body macrosomatognosia and microsomatognosia and the dead-weight hallucination that was mentioned before. Another artist did not intentionally paint distortions based on her own perceptions but deserves mentioning because of the unusual nature of her dreams. She experiences a dream dynamic that is best described as a complex nocturnal Frégoli syndrome. During dreaming she jumps into all characters in her dream, seeing the unfolding scene from multiple perspectives. For example, she hears the character next to her speak, and will then be the person speaking. Her work depicts multiple overlapping portraits producing a sense of double vision or triple vision. It is interesting to note that in her dreams this artist experiences the dream from the perspective of multiple characters and her painted portraits have multiple pairs of eyes (**Figure 14**).

### 4.3 Relation with self-reported medical conditions

Regarding the relation of distortions in paintings to underlying medical conditions, the majority of our participants self-reported having no medical diagnosis. Exceptions included one person who had been hospitalized for major depressive disorder, and one awaiting surgery for a cervical nerve-sheath tumor. Including these two people, five participants mentioned having experienced anxiety, panic attacks or extreme stress, albeit without mentioning whether this had resulted in a psychiatric diagnosis. As indicated above, nine participants suffered from migraines. The six among them who experienced migraine with aura reported on distortions such as polyopia, hypoacusis, hyperchromatopsia, tunnel vision, and illusory visual spread. Moreover, 13 people experienced some sort of sleep disorder, such as hypnagogic or hypnopompic hallucinations, sleep paralysis, persistence of dream imagery into waking, REM sleep disorder, insomnia and night terrors. Of our participants, 55% had either been prescribed a medication that appeared to have caused distorted perceptions or had experimented at least once with a psychotropic drug (i.e. marijuana, LSD, mushrooms). It would have been interesting to see whether the neurological conditions reported on might be associated with distortions that differed in some way from those associated with non-neurological conditions, but a preliminary analysis on the (limited number of) data here collected did not indicate that this was the case.

#### 4.3.1 Other sources of inspiration

We also investigated whether artists were inspired by distortions in the artwork of others, or by glitches in digital images, or that they used technology to intentionally distort images, whether or not for commercial gain. One artist mentioned specifically that the distorted faces in his paintings are a direct influence from Francis Bacon’s work. Another artist mentioned studying glitches in photographs and videos to inspire his work. In fact, three artists mentioned using technology to distort images or inspire distortions in their painting. Two artists mentioned being inspired by neuroscience, one even titling a piece with facial distortions *Prosopometamorphopsia*.

### 4.4 Prevalence rates

Compared to the information that is available on the prevalence of positive disorders of perception in the general population, the rate of 85% that we found may strike one as rather high. However, in a large online survey among 10,448 people in the general population, Linszen et al. (21) found a lifetime prevalence of 80% for hallucinations and a limited number of other misperceptions. This is much higher than the rate of 4-21% that has been reported in ten large-scale epidemiological studies on hallucinations throughout the past century and a half (22). One may ask oneself whether the high rates found by Linszen et al. have something to do with selection bias, in the sense that people who hallucinate may more eagerly participate in such an online survey than others, but it may also be an effect of the rather wide scope of their survey, in contrast to traditional epidemiological studies that tend to limit the range of phenomena under study to four or five types of hallucination. As a consequence, it may well be that in any group of randomly selected people in the general population, careful interviews will also yield prevalence rates of around 85% for perceptual distortions, hallucinations, and other perceptual phenomena such as we found in our group of artists. As noted above, studies of perceptual distortions in nonclinical populations in both adolescents (17) and adults (18) indicate that metamorphopsias are relatively common. Thus Lipsanen et al. found that 38% of their participants experienced metamorphopsias, even though their study protocol was quite narrow and participants were specifically requested to disregard distortions experienced under the influence of drugs or alcohol. The four (out of 40 known) metamorphopsias that they asked about were macropsia, micropsia, teleopsia (seeing things further away) and dysmorphopsia (seeing lines as wavy). If we apply the same criteria to our interview data, removing all metamorphopsias attributed by the participants to pharmacological causes, five out of our 20 participants, or 20%, fit the criteria used by Lipsanen et al., compared to 55 out of 297 people (18.5%) in their study.

### 4.5 The artists

Future investigation could probe whether artists in general are more likely to experience distorted perceptions, or if this particular subgroup of artists who paint distorted images are more likely to experience distortions than non-artists. However, on the basis of the prevalence rates reported above, it might also be that anomalous perception is much more common in the general population than studies have traditionally revealed, and that these artists merely use their experiences in this area to their advantage. The majority of our artists didn’t create work inspired by the distortions they themselves experienced. This presents the possibility that artists experiencing any sort of distortion to perception may be more open to rendering distortions in their work. In other words, if they have experienced occasions when objective reality can be called into question, maybe their representative artwork can also deviate from the expected. Despite most artists not portraying their experienced distortions in their work, it is of interest that the majority of the phenomena reported were visual in nature. This complies with the distribution of distorted perceptions in AIWS, although here it could also be a reflection of our target population. After all, since all participants were visual artists, they may have been more attuned to visual stimuli.

### 4.6 The appeal of disrupted realism

If it is true that anomalous perception is much more common than traditionally recognized, this may give even more weight to our assumption that people are fascinated by such paintings because their brain recognizes something while they themselves hardly realize that they experienced it. Elaborating on this theme, the appeal of disrupted realism may also lie in the fact that distorted images are part and parcel of regular visual perception, for example when images are reflected in curved objects such as a metal bowl or the hood of a car, when they are seen through irregular surfaces such as water or a stained-glass window, or when they are seen through layers of hot and cold air above a road or a desert. Such physical illusions fall into a class of their own, but phenomenologically they come close to the perceptual distortions characteristic of AIWS. Another explanation for the appeal of disrupted art may be that people recognize something they experienced while ill. This goes both ways, with artists recreating what they perceived while ill, and other people recognizing that. Regardless of the reasons underlying the distorted work, these pieces remain intriguing and interesting to view. Neurological disorders may underlie the unique perspective of a particular artist, like the work of Francis Bacon with prosopometamorphopsia or Chuck Close who was known to have prosopagnosia (i.e. the inability to recognize faces). Looking at the artwork only as a manifestation of disease would miss much of what art offers, namely an exchange between the artist capturing their unique personal perception and sharing it with a broader audience, allowing the viewer a glimpse of the world through another’s eyes. Perhaps artwork that depicts perceptual distortions may help to bridge this gap and foster understanding of experiencing distorted perceptions.

### 4.7 Angor animi as a type of complicated metamorphopsia?

As mentioned above, one participant had experienced an overwhelming sense of impending doom preceding a cardiac infarction. In her own words, the infarction had been caused by a viral infection, two days prior to her 30th birthday. She recalled, ‘I just knew I was dying.’ This sense of impending doom can be characterized as a complicated metamorphopsia, i.e. a perceptual distortion that is accompanied by an alteration in the affective tone of one’s assessment of the environment (3, 23) - in this case, rendering it dark and foreboding. This particular type of complicated metamorphopsia is known in the cardiological literature as angor animi (24, 25). Todd, in his original paper on AIWS, captured the myriad of metamorphopsias experienced by his patients, including angor animi, as described by a 32-year-old woman with ‘bizarre symptoms’:

*She was also troubled by recurrent sensations that she was about to die; these attacks of angor animi, which lasted about half an hour, were accompanied by an illusory slowing in the passage of time* (2).

As Todd concludes, ‘In this case, the disorders of body image, metamorphopsia, angor animi, etc., appear against a background of migraine-epilepsy.’ Although the majority of citations of angor animi are associated with angina pectoris, often preceding other cardiological symptoms, it can also precede an epileptic seizure or occur in the context of a migraine prodrome. Categorizing angor animi as a complex metamorphopsia that falls within the scope of AIWS, as Todd did, has the benefit of engendering interdisciplinary conceptualization and facilitating differential diagnosis, though caution must be exercised in considering the case of angor animi reported by our participant as an AIWS symptom since it was experienced only once in conjunction with a cardiac infarction and at the time of the interview had not recurred.

### 4.8 Limitations

Our study has several limitations. As far as we know this is the largest study on AIWS among artists, but nonetheless the sample size was relatively small. Therefore, extrapolation of our findings must be done with reservations. Moreover, there was no control group and the questionnaire that we used was not validated. Although several thorough epidemiological studies exist on hallucinations in the general population, we had no similar studies on perceptual distortions to compare our results with. The three artists who returned their questionnaire via email reported very few or no distorted perceptions. Perhaps this captures a fundamental difference in the detail and number of data captured via interview as opposed to a written questionnaire or the participant’s level of willingness to participate. Another limitation is that we have no explanation for the relatively high number of perceptual distortions found in individual participants. That said, the SIntAD, our tailor-made semi-structured questionnaire, may have helped us to capture more symptoms of AIWS. Previous studies have focused on a narrower group of symptoms or excluded symptoms caused by drug use, while we chose to include all currently known symptoms of AIWS from all causes. Finally, the links suggested with medical conditions throughout our article cannot be taken at face value, since they were self-reported conditions that we did not diagnose ourselves. Neither did we have access to our participants’ medical files to see what diagnoses had been established by other health professionals.

### 4.9 Conclusion

Our study among 20 painters in the disrupted-realism movement indicates that the distortions incorporated in their work rarely represent their own, lived-through experiences. Nonetheless 55% reported being aware of having experienced perceptual distortions, and 85% having experienced positive disorders of perception in general. Whether this is a large proportion in comparison to the general population is hard to say due the lack of large-scale epidemiological studies on perceptual distortions. Indirect evidence does indicate though, that the prevalence rate among these painters may not be exceptional, although their ability to utilize distortion as a means to render unique and fascinating impressions of their world may be. Experiencing perceptual distortions may allow the artists a certain flexibility to deviate from reality when representing the objects in their paintings, but it is by no means a given. The three artists in our sample who had no unusual perceptions to report still created exceptional artwork containing intriguing distortions. Since all of our participants who reported migraine with aura also experienced AIWS symptoms, this could explain our finding a higher prevalence of AIWS symptoms. Circling back to Plato, our study indicates that the Greek philosopher’s theory of mimesis does indeed take on a special meaning in the context of Alice in Wonderland syndrome. When artists experience perceptual distortions and employ these - or variants thereof - in their work, can we then say that the result is farther removed from Plato’s eternal, unattainable truth than a painting in an orthodox realist style? Or is it rather closer to the truth, since it incorporates as faithfully as possible that what the artist perceives? Whatever the answer to that question may be, distorted realism offers an opportunity to share an artist’s unique personal rendition of reality and perhaps also recognize, consciously or subconsciously, distortions that one has experienced oneself.

## Data Availability

The original contributions presented in the study are included in the article/**Supplementary Material**. Further inquiries can be directed to the corresponding author.

## Supplementary material

Supplementary Table 1 Disorders of perception and associated medical conditions, as reported on by 20 artists.

Supplementary Table 2 Structured Interview for Artists using Distortions in Painting (SIntAD).
