# Supplementary material for: Perceptual distortions characteristic of Alice in Wonderland syndrome in contemporary figurative painting
Source: Front Psychiatry. 2024 Dec 4;15:1466666. doi: 10.3389/fpsyt.2024.1466666 (PMC11652831; doi:10.3389/fpsyt.2024.1466666)
Supplement: Supplementary Table 2 — Structured Interview for Artists using Distortions in Painting (SIntAD). [file Table2.docx]

Supplementary Material Structured Interview for Artists using Distortions in Painting (SIntAD)

All of your responses are confidential, and data is aggregated to ensure your anonymity. If you don’t understand a question or something seems ambiguous, please ask me for clarification. Feel free to take your time and think about your answers.

I’ll be asking you a number of questions about you and your artwork and about things that may influence your perceptions or your senses. This part of the questionnaire should take about 15 minutes. Do you mind if I record our conversation so that I have an accurate record of your answers?

1. Are you aware that your artwork is distorted or contains distortions from reality?
2. Are the distortions intentional?
   1. If they are not intentional, how do they arise?
   2. If the distortion is intentional, can you tell me about the reason for the distortion?
3. Is your painting an accurate portrayal of the way you see the world? In other words, is this painting an accurate depiction of your perceptions?
   1. If this is not an accurate portrayal of your perceptions, is it stylized? Can you explain how it is stylized?
   2. Is there something else that you are portraying with your choices? Some examples could be an emotion, a person’s character, movement of time, a feeling.
4. Are the distortions that you depict based on your personal experience?
5. Are distortions in your work symbolic? If yes, can you tell me about the symbolism?
6. Are some distortions in your work perceptual; i.e.. based on something you have felt, seen or have experienced?

The next set of questions cover conditions that can alter people’s perceptions. You can skip a question if you prefer not to answer, but keep in mind that your answers are confidential, and results are aggregated which preserves your privacy.

1. Participant history

Have you had a high fever that caused hallucinations or unusual sensations?

Have you had sleep deprivation that has caused visual hallucinations?

Have you taken hallucinatory drugs or been prescribed medication that may alter perceptions?

Do you have any sleep related disturbances, like sleep paralysis or seeing, feeling, or hearing something that isn’t there as you’re falling asleep or waking up?

1. Do you have any other experience of hallucinations or unusual perceptions that I haven’t asked about? Can you tell me a bit more about them?
2. May I use images of your artwork as examples in my paper? The paper will be submitted to an academic journal for publication. If you agree, I’ll send you a form for permission to use specific images that you can sign and return to me.
3. Would you like to receive a copy of the paper when it’s published?

Are there any questions that you have for me?
