# Supplementary material for: Perceptual distortions characteristic of Alice in Wonderland syndrome in contemporary figurative painting
Source: Front Psychiatry. 2024 Dec 4;15:1466666. doi: 10.3389/fpsyt.2024.1466666 (PMC11652831; doi:10.3389/fpsyt.2024.1466666)
Supplement: Supplementary file 2 [file Table1.docx]

Table S1

Subject Number	Sex	Age Range	AIWS Specific Symptoms	Hallucination	Other unusual perception	Possibly relevant diagnosis
1	F	51-60			Sleep paralysis upon waking	
				Sleep deprivation hallucinations		
2	F	51-60	Total body microsomatagnosia			
			Total body macrosomatagnosia			
					Syneaesthesia	
			Hyperchromatopsia migraine			
			Hypoacusis - migraine			
3	M	61-60	Gyropsia with drug use			
			Hyperchromatopsia drug use			
				Complex hallucinations with illness and fever		
				Hallucination of void/abyss with extreme stress		
4	F	31-40				
5	M	51-60	Microsomatagnosia			
			Macrosomatagnosia			
				Dead-weight hallucination		
6	M	41-50	Enhanced stereoscopic vision			
				Hallucinations with fever		
7	F	61-60	Depersonalization			
				Auditory hallucination		
					Grapheme-color synaesthesia	
						Duane's syndrome
8	M	41-50			Syneaesthesia	
					Out of body experience	
				Hallucination during meditation retreat		
			Dysmorphopsia - LSD			
			Time distortions - ayhuasca			
9	F	61-60			Chromesthesia or sound to color synesthesia	
				Grief hallucination		
				Hallucination of imagery with fever		
10	M	41-50		Hypnagogic hallucinations		
11	F	51-60				
12	M	31-40				
13	F	21-30			Hypnic jerks	FND
				Hypnopompic hallucinations		
					Severe Nightmares	
				Sleep deprivation hallucinations		
			Derealization - psychosis			
			Dysmorphopsia - migraine			
			Hyperchromatopsia - migraine			
14	F	21-30	Polyopia			
			Auditory hallucinations			
			Depersonalization			
			Enhanced stereoscopic vision			
15	M	41-50			He touches his subject with his eyes when he paints them - Synesthesia	
16	M*	21-30				Nerve sheath tumor between C1 and C2
					Hyperacusis	
				Lilliputian hallucinations with fever		
					REM sleep behavior disorder	
					Insomnia	
					Night terrors	
			Hyperchromatopsia			
			Enhanced stereoscopic vision			
			Tunnel vision			
17	M	31-40		Hypnopompic hallucinations		
					Sleep paralysis	
					Talking in sleep	
					REM Sleep Behavior Disorder	
				Visual & Auditory hallucination with flu and medication		
			Tunnel vision			
			Mosaic vision			
			Hyperchromatopsia			
			Visual preservation/trailing effect			
18	M	31-40	Hyposchematia			
					Extremely vivid dreams	
					Insomnia	
			Visual preservation/trailing effect			
19	M	41-50			Sleep paralysis	
				Night hag hallucination		
					Enhanced stereoscopic vision	
					Hyperchromatopsia	
					Time distortions	
				UFO hallucination		
					Highway hypnosis - time gap experience	
					Enhanced stereoscopic vision	
				Fever hallucinations - visual and somatic		
			Gyropsia			
20	F	41-50		Hypnopompic hallucinations		Myocardial infarct
					Unusually vivid dreams	
					Complex Fregoli syndrome	
			Complicated metamorphopsia - sense of impending doom prior to heart attack			

** Biological male identifying as female*

Color key
Unspecified cause
Migraine
Pharmacological
Sleep related
Fever
Meditation
Grief
Stress
Psychosis
Heart infarct
